# MicroStretch: Microstretcher designed for live imaging on microscopic stages

**DOI:** 10.1016/j.ohx.2025.e00737

**Published:** 2026-01-03

**Authors:** Alexi Switz, Anamika Prasad

**Affiliations:** a Biomedical Engineering, Florida International University, Miami, FL, United States; b Mechanical and Materials Engineering, Florida International University, Miami, FL, United States

**Keywords:** Micro Stretcher, Strain, Mechanical performance, Imaging, PDMS, Electrospun fibers

## Abstract

Deformation response evaluation is essential for understanding material behavior, providing insight into their suitability across many fields, such as biomechanics, materials science, and other engineering disciplines. Specialized applications in biomedical and soft materials demand miniaturization for testing under a microscope or spectroscopic stages. The current commercial machines on the market are often large, expensive, or heavy, making them difficult to use for specific needs. This hardware addresses this need by developing a cost-effective, miniature, and programmable system that can be tailored to individual lab requirements to fit multiple microscopic stages. By utilizing a bipolar stepper motor attached to a lead screw and sliding linear stage, programmed and controlled by an Arduino microcontroller, the system can apply specialized stretch under uniaxial static or cyclic loading. The developed system can be assembled for less than $100, making cost-effectiveness a central focus of this development. The device performance was validated using a variety of samples and microscope tests, with sample deformation captured in real time. The device is compatible with live imaging on microscopic stages, accommodating specialized research needs across applications.

## Highlights

• Design and manufacturing of an affordable, easy-to-assemble micro stretching system for use on microscope stages.
• Hardware is compact and lightweight, making it versatile for use on a variety of microscope stages.
• Equipment is validated using PDMS and electrospun samples stretched and real time imaged on multiple microscopes.
• Detailed CAD models, assembly processes, and parts lists provided for easy replication and modification.
• Open-source innovation democratizes the use of important testing approaches.

## Graphical abstract

**Figure:**
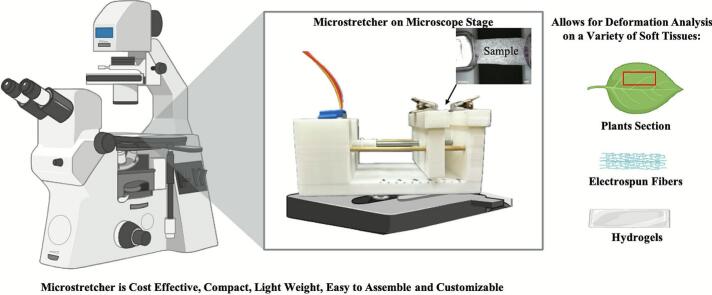

**Specifications Table:**

**Table:** 

Hardware name	RL Micro-stretcher
Subject area	Material Science
Hardware Type	Programmable deformation stage for imaging
Closest Commercial Analog	Strex Cell Manual Stretch Device (ST-0040)
Open Source License	This work is licensed under a Creative Commons Attribution 4.0 (CC BY 4.0) International License.
Cost of Hardware	<$100
Source File Repository	https://data.mendeley.com/datasets/74jmhyd2g7/1

## 1 Hardware in context

Miniature tensile testing is an important tool for understanding the behavior of materials, providing valuable information across many disciplines, such as additive manufacturing [1], biomedical [2], and polymer science and nanocomposites [3]. Tensile test miniaturization typically addresses the need for material testing of small or soft samples, which otherwise could not be fitted on a standard stage. In other cases, the need can be driven explicitly for in-situ visualization of localized failure and deformation mechanisms [1,4,5] or for quantifying strain through bond shift analysis under Raman spectroscopy [6,7]. Here, the need for in-situ visualization drives the development of a cost-effective miniaturized platform capable of applying defined stretches and the setup capable of being integrated on standard optical imaging and spectroscopic stages.

Many commercial miniature tensile testers are available on the market; however, they are not widely accessible due to cost or size restrictions, or both. Other expensive commercial stretchers, such as Flexcell (USA), are specifically designed for cell stretching but are outside the focus of this development as they require specialized bioreactor setup for maintaining the physiological environment. Commercial microstretcher options that do not provide force measurements and are capable of fitting on a microscope stage such as the Strex Cell ST-0040 or ST-0100; are limited to manual power, maximum 20 % strain and cost over $1,300. Strex Cell also offers an automated microstetcher; however, its price is not publicly available online. There is, overall, a lack of commercial setup packaged for both imaging and tensile stretch at a cost-effective range and customizable for user’s needs for wide-scale access across the scientific community.

In response to this need, multiple laboratories have developed miniature tensile testers with tailored designs to meet their specific testing needs [8,9,10,11,12,13,14,15,16,17,18,19]. While each group successfully produced a functional micro-tensile tester, none of the existing designs are both compact enough to fit on a microscope stage and cost-effective, which is the focus of the current development. Most lab-built tensile testers remain bulky, expensive, or incompatible with integration into advanced microscopic imaging systems. To conduct live imaging analysis of samples, many labs have also developed in-house micro-stretcher hardware to test either nanomaterials or cellular responses to applied strain; however, few provide significant details about the system for replication [20,21,22]. Other groups have developed specific microstretchers to accommodate live imaging of cells undergoing strain; however these devices are not compatible with testing of non-cellular culture samples [23,24,25,26,27,28,29]. Shiwarski, et. al developed a $580 biaxial microstretcher with temperature controls to allow for live fluorescence cellular imaging [30]. Mayer, et. al developed a uniaxial microstretcher; however this device is manually powered [31]. These developed microstretchers remain either costly, too large or incompatible with automated uniaxial biomaterial testing.

A need for a cost-efficient (<$100), small enough to fit on a microscope stage (20 cm x 15 cm x 7 cm), with a tunable range of strain rate and fully customizable stretcher is still apparent. Here, we address this need by developing a miniaturized, fully programmable setup that fits a standard-sized microscopy stage and costs less than $100. A comparison of the commercially available microstretchers to our in-house microstretcher is shown in Table 1. The decision to focus on the application of defined strain was guided by the primary need of miniaturization for integration with imaging tools for the study of localized deformation and failure mechanisms, as noted earlier. The development of a miniature stretcher was approached by utilizing a bipolar stepper motor attached to a lead screw and a sliding linear stage, programmed and controlled by an Arduino microcontroller. The stretcher was demonstrated to be compatible with multiple imaging stages, such as digital and confocal microscopes.

**Table 1:** Comparison of Commercial alternatives to in-house developed Microstretch.

Hardware Name	**Microstretch** (current device)	**ST-0040**	**ST −1500**
Manufacturer	Prasad Lab	Strex Cell	Strex Cell
Tupe	In-house	Commercial	Commercial
Cost of Machinery	<$100	$1,337	Not Listed Online
Dimensions	17 cm × 13 cm × 5 cm	Not Listed Online	Not Listed Online
Powered	Automated	Manual	Automated
Force Measurements	N/A	N/A	N/A
Maximum Strain	300 %	20 %	20 %

## 2 Hardware description

The developed tensile stretcher focuses on being low-cost, miniature, programmable, and designed for use under a microscope or in a spectroscopy setup. The total device dimensions are 12 cm × 17 cm × 6 cm; however, the orientation of the breadboard and Arduino can be adjusted to better fit different microscope stages. The maximum theoretical force capacity of the device is close to 270 N and displacement accuracy of 0.39 μm (see supplementary information for details).

**Fig. 1:**
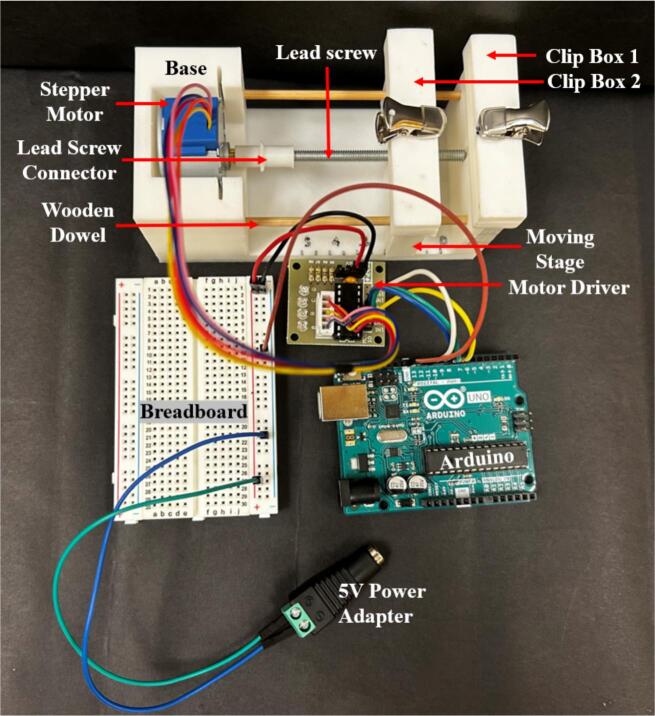
Labeled diagram of microstretcher hardware.

As shown in Fig. 1, it is composed of a base made from 3D-printed PLA (polylactic acid) filament, with a lead-screw-driven moving stage actuated by a bipolar stepper motor. Clip box 1 is fixed and attached to the base, while clip box 2 is mounted on top of the moving stage for stretching. The motor is controlled using an Arduino-based circuit with a motor driver and powered using a 5 V wall converter. The programming is done in Arduino (codemicrostretcher.ino), making it free and easy to use. The desired stretch can be typed into the Arduino prompt. Speed can be changed using the calibration table and adjusting “my stepper speed” (line 7) in the code.

The Arduino code uses the ‘Stepper’ library to control the bipolar stepper motor and begins by establishing the motor control pins (8, 9, 10, and 11). In the main loop, the sketch prompts the user to enter the desired movement distance in millimeters in the serial monitor. This value is converted to motor steps, and the motor moves forward. Once the opening distance is achieved, the user is prompted to enter a closing distance, and the motor moves in the opposite direction of the first distance input. The tensile stretcher described offers full programmability, compatibility with microscopy and spectroscopy, and the ability to be customized further to meet user needs. This device applies controlled displacement for imaging applications. It does not measure force and therefore cannot determine mechanical properties such as modulus or strength. For applications requiring quantitative mechanical property data, this device would need to be integrated with or adapted to include force measurement systems.

## 3 Design file summary

All the files listed in Table 2 below are available on Mendeley at: https://data.mendeley.com/datasets/74jmhyd2g7/1.

**Table 2:** The table detailing the files used to assemble the Microstretcher, with the location of the file provided.

Design file name	File type	Open source license	Location of the file
Base	CAD & STL	CC By 4.0	https://data.mendeley.com/datasets/74jmhyd2g7/1
Clip_Box_1	CAD & STL	CC By 4.0	https://data.mendeley.com/datasets/74jmhyd2g7/1
Clip_Box 2	CAD & STL	CC By 4.0	https://data.mendeley.com/datasets/74jmhyd2g7/1
Motor_to-lead_screw_connector	CAD & STL	CC By 4.0	https://data.mendeley.com/datasets/74jmhyd2g7/1
Moving_Box	CAD & STL	CC By 4.0	https://data.mendeley.com/datasets/74jmhyd2g7/1
Arduino Code codemicrostretcher.ino	IDE	CC By 4.0	https://data.mendeley.com/datasets/74jmhyd2g7/1

## 4 Bill of materials summary

The bill of materials is provided in Table 3 below with links for purchase. The total cost is < $100. The cost of a 3D printer is not included in the list, but printing can be done using a cost-effective 3D printer or other alternative additive manufacturing methods readily available. We have chosen to print using PLA as linked in Table 3 for its ease of use, low weight, low cost, and wide commercial availability; however, alternative materials will work in substitute.

**Table 3:** Materials used to assemble the platform and their cost, with link. The total cost of materials comes to 95.62 USD.

Designator	Component	Qty	Cost per unit − USD	Total Cost − USD	Source of Materials	Material Type
Arduino (Arduino Uno REV3 [A000066])	B008GRTSV6	1	$27.60	$27.60	Amazon Link	Electronics
Arduino Cord (3M Arduino UNO USB)	B08RCJXY1Z	1	$7.99	$7.99	Amazon link	Electronics
Breadboard and Jumper wires	B08Y59P6D1	1	$9.99	$9.99	Amazon Link	Electronics
5 V Power Adapter	B0DQW6HD5C	1	$8.29	$8.29	Amazon Link	Electronics
Filament (Anycubic Filament 1.75 mm)	B083BQTGFX	1	$11.99	$11.99	Amazon Link	Polymer
Rod (8–32 x 1ft)	# 2294	1	$1.36	$1.36	Home Depot Link	Metal
Motor (ELEGOO 28BYJ-48 ULN2003 5 V Stepper Motor + ULN2003 Driver Board)	EL-SM-003	1	$14.99	$14.99	Amazon Link	Electronics
Clips (30 PCS Mini Metal Alligator)	564730_1_TAX2KmNR5	1	$8.85	$8.85	Amazon Link	Metal
Wooden Skewers (12-inch bamboo skewers)	076753244558	1	$4.56	$4.56	Amazon Link	Wood

## 5 Build instructions

1. Print all STL files on a 3D printer using PLA filament. The files were printed on an Ankermake M5 printer, using a 0.4 mm Nozzle at a nozzle temperature of 200 °C, a printing bed temperature of 70 °C and a printing speed of 150 mm/sec. Printing parameters may need to be adjusted depending on 3D printer performance.
2. To make the measurement markings more visible on the base, use a black sharpie to outline their indentations as shown in Fig. 2, Fig. 2.

**Fig. 2:**
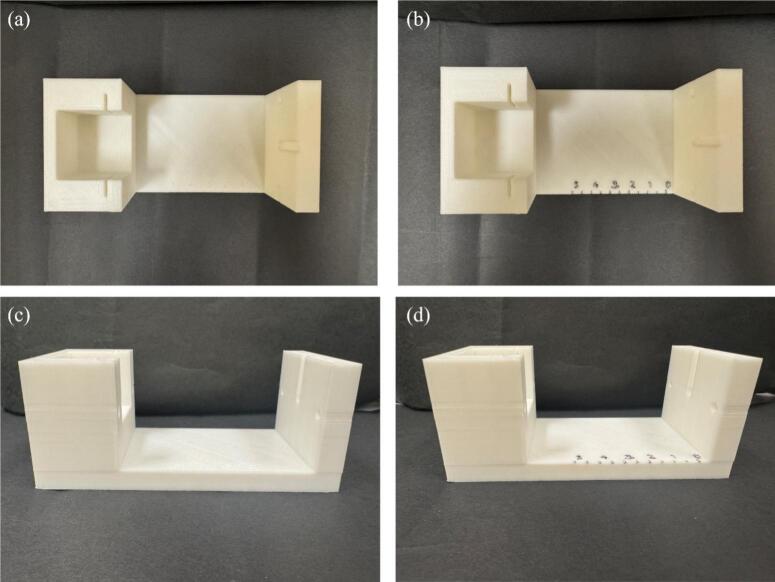
PLA printed part Base in top and side view with outlined measurement markings.
3. Construct the circuit on a breadboard using a ULN2003 stepper motor driver using the steps below.
a. Attach the 5-pin connector from the 28BYJ-48 stepper motor to the ULN2003 driver, as shown in Fig. 3a.

**Fig. 3:**
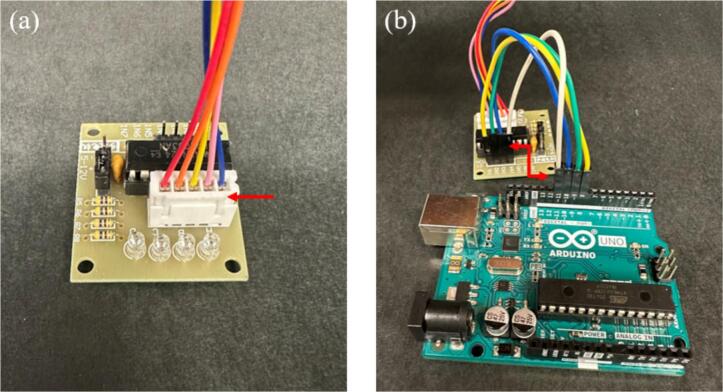
(a) 5-pin connector from 28BYJ-48 stepper motor attached to the ULN2003 stepper motor driver, and (b) Driver’s input pins IN1, IN2, IN3, and IN4 connected to Arduino UNO pins 8, 9, 10, and 11, respectively. An arrow is drawn to mark the connections.
b. Connect the motor driver’s input pins to the Arduino UNO using female-to-male jumper wires. Specifically, connect IN1, IN2, IN3, and IN4 to Arduino pins 8, 9, 10, and 11, respectively, as shown in Fig. 3b.
c. Connect the positive and negative terminals of the motor driver to the breadboard’s power rails, as shown in Fig. 4a.

**Fig. 4:**
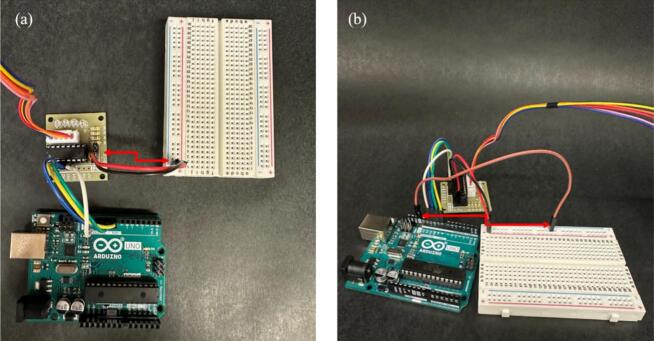
(a) Positive and negative terminals of the motor driver connected to the breadboard’s power rails, and (b) Ground wire from the negative screw terminal of the motor driver connected to the Arduino’s GND. Arrows are drawn to mark the connections.
d. Connect a ground wire from the negative screw terminal of the motor driver to the Arduino's GND pin, as shown in Fig. 4b.
e. Power the stepper motor using a 5 V 2A wall adapter, connected via a female DC jack adapter with jumper wires [32], as shown in Fig. 5a

**Fig. 5:**
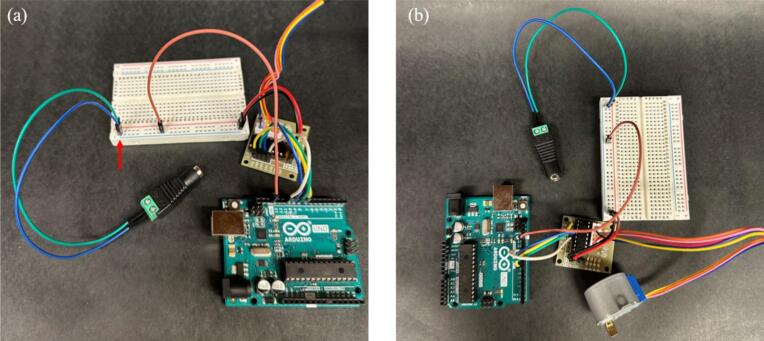
**(a)** Stepper motor connected via a female DC jack adapter with jumper wires, and (b) Full setup of the Arduino connections and constructed circuits. An arrow is drawn to mark the connection.

The complete setup for step 3 is shown in Fig. 5b.

4. The lead screw rod was manufactured from a 12-inch-long, 0.25-inch-diameter threaded rod (Home Depot). The rod was cut into a 2.75-inch-long piece using a hand saw, and the edges were sanded to ensure the stage could grip onto the threads.
5. Using a small hand saw, cut a wooden dowel into two 3.75-inch-long sections. These pieces serve as side support rails for the device.
6. Use glue to adhere a clip on top of each printed Clip_box part at the midpoint as shown in Fig. 6. These clips will act as holders for the samples to be tested.

**Fig. 6:**
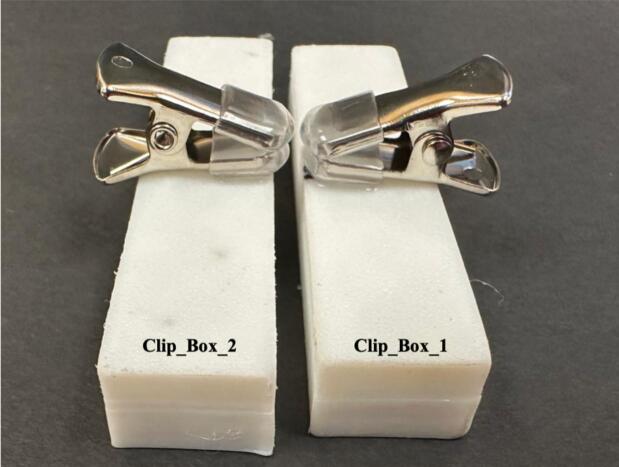
Clip holders attached to the Clip_Box printed part for sample testing.
7. Insert the lead screw into the lead screw connector and attach it to the motor as shown in Fig. 7a.

**Fig. 7:**
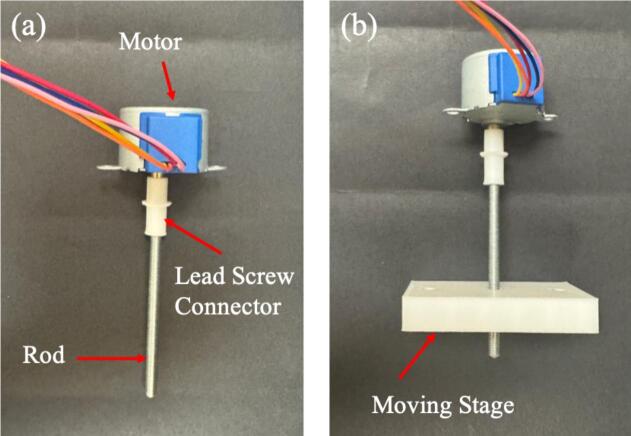
Lead screw attached to the motor connector and set inside the moving stage.
8. Insert the lead screw into the corresponding opening in the moving stage by gently twisting clockwise as shown in Fig. 7b. When the lead screw is rotated, the part should glide smoothly, demonstrating the functionality of the mechanical design.
9. Place the motor in the base compartment, ensuring the wires face upwards (Fig. 8 and Fig. 9a). The compartment is designed to hold the motor in place securely; however, if the motor moves, secure it in place using adhesive.

**Fig. 8:**
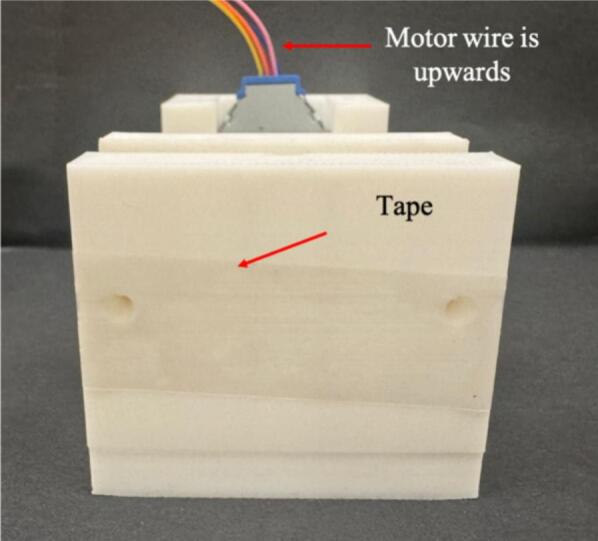
Wooden dowels secured to the base using tape.

**Fig. 9:**
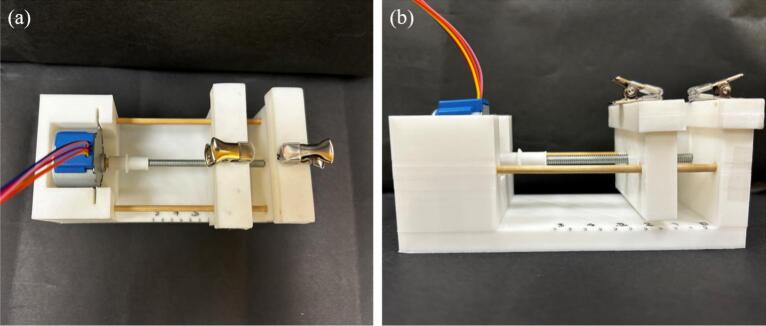
Assembled microstretcher in two views a) top view b) side view.
10. Insert the wooden dowel side rails into the outer holes of the base and the moving box (Fig. 9). Secure the wooden dowels in place with tape as shown in Fig. 8.
11. The two printed clip boxes can now be placed, with clip box 1 on top of the base stage and clip box 2 on top of the moving stage. The assembled micro-stretcher system is shown in Fig. 9 in two different views.
12. Connect the system to the Arduino using the female wire connectors. The connection is shown in Fig. 10.

**Fig. 10:**
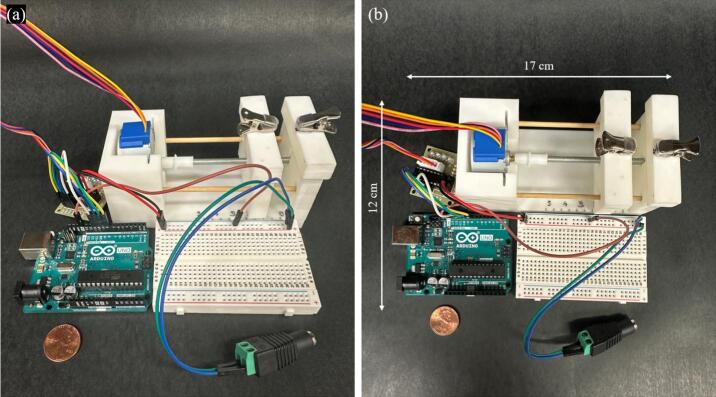
Assembled Microstretcher connected to Arduino controls and power in two views a) side view b) top view.

## 6 Operation instructions

1. Open the Arduino software and code, then plug the Arduino into the computer's USB port.
2. Use the calibration table to determine the desired strain rate and then adjust line 7 in the code to match the desired speed.
3. Upload the code to the Arduino.
4. Measure the length of the sample and enter the sample length into the Arduino code. The clamp will move to slightly less than the length of the sample. The extra sample length will be inside the clamps.
5. Clamp the sample into the clips.
6. The code will prompt for the closing distance, type in zero, typing in an integer will compress the sample.
7. Type in the desired stretch length for the sample. The moving box will move, and measurements/ analysis of the sample can be taken during and after the stretch.
8. If the test needs to be stopped at any time, simply type s into the command prompt. This will terminate the test and stop the motor.
9. For additional stretch cycles, type in additional stretch/ compression distances.
10. When testing is finished, remove the sample from the clamps and type in the closing distance.

Potential safety hazards for this device include the use of electrical components. It is important during assembly to ensure proper grounding and connection of all circuitries. It is important when operating the device to ensure all body parts including hair are kept clear of the motor and rod to prevent injury. While operating the device, if any safety concerns arise and the test needs to be stopped, ‘s’ can be typed into the command prompt to terminate the test. In testing various materials, ensure proper safety precautions are used based on sample specific requirements.

## 7 Validation and characterization

### 7.1 Size compatibility with microscope stages

**Fig. 11:**
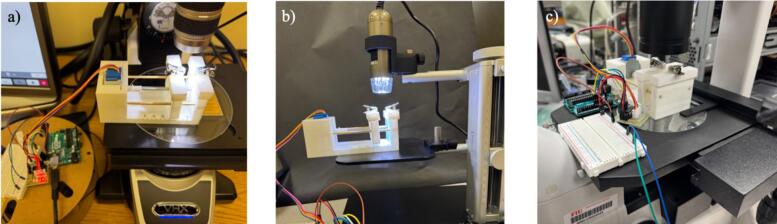
Microstretcher on microscope stages a) Keyence Digital Imaging, b) Dinolite Handheld microscope, and c) Amscope cell imaging microscope.

To validate the hardware’s size compatibility with various microscope stages, the hardware was placed on three microscopes: 1) Keyence Digital Imaging, 2) Dinolite Handheld microscope, and 3) Amscope cell imaging microscope. As shown in Fig. 11, the micro stretcher can fit on each stage.

### 7.2 Strain rate calibration

**Fig. 12:**
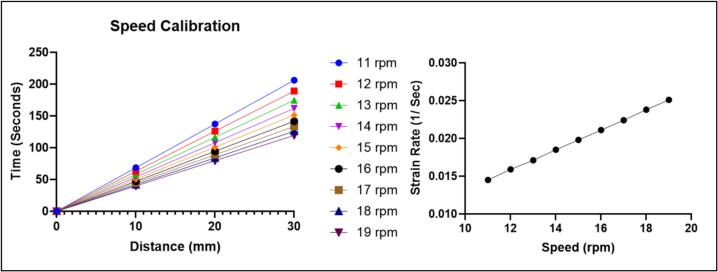
Plot of speed calibration (see supplemental information Table 1, Table 2, Table 3 of full data).

To determine the stretcher’s strain rate, we timed how long it took the stretcher to displace 10 mm, 20 mm, and 30 mm. Total distance traveled was recorded using a ruler for each trial. In each trial the total distance traveled was identical to the input distance in the code. The timing was recorded using a stopwatch built into the code. We repeated each measurement ten times and at multiple stepper speeds (11, 12, 13, 14, 15,16, 17, 18, and 19 rpm). Strain rate was then calculated by dividing strain (change in length divided by original length) by time. All average values, corresponding standard deviations and strain rates are shown in Table 4 and Fig. 12, with full data provided in Supplementary information, tables S1 to S3.

**Table 4:** Calibration table average time and speed.

**Stepper speed (rpm)**	**Average 10 mm time (sec)**	**Standard deviation**	**Average 20 mm time (sec)**	**Standard deviation**	**Average 30 mm time (sec)**	**Standard deviation**	**Strain rate (1/sec)**
11	68.73	0.003	137.47	0	206.21	3.00*10^−14^	0.0145
12	63.05	0	126.11	0.005	189.16	3.00*10^−14^	0.0159
13	58.23	0.011	116.47	0.016	174.7	0.021	0.0171
14	53.97	1.50*10^−14^	107.94	3.00*10^−14^	161.92	3.00*10^−14^	0.0185
15	50.46	7.50*10^−15^	100.92	0.011	151.38	0.011	0.0198
16	47.28	7.50*10^−15^	94.56	1.50*10^−14^	141.84	3.00*10^−14^	0.0211
17	44.48	7.50*10^−15^	88.97	1.50*10^−14^	133.46	0.005	0.0224
18	42.02	7.50*10^−15^	84.04	1.50*10^−14^	126.07	3.00*10^−14^	0.0238
19	39.86	7.50*10^−15^	79.71	0.004	119.57	0.003	0.0251

### 7.3 Sample stretching with live imaging

To validate the hardware’s ability to stretch samples, the hardware was tested using a standard PDMS Sylgard 184 (Millipore Sigma, 761036-5EA) sample and polycaprolactone electrospun fibers. The PDMS was made using a 1:10 ratio of curing agent to polymeric base. The solution was cured in water-soluble PVA molds overnight at 65℃. The molds were then dissolved away, leaving the PDMS samples in the ISO 527 2 Type 5B shape. The fibers were produced using a methodology previously described [33]. A 15 mm × 5 mm section of electrospun nanofibers was used for each test.

**Fig. 13:**
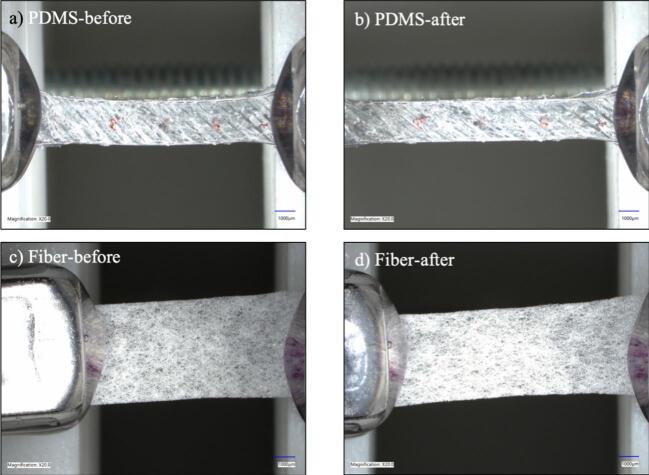
Demonstration under a Keyence microscope of sample stretched at 11 rpm for (a and b) PDMS and (c and d) electrospun fibers.

**Fig. 14:**
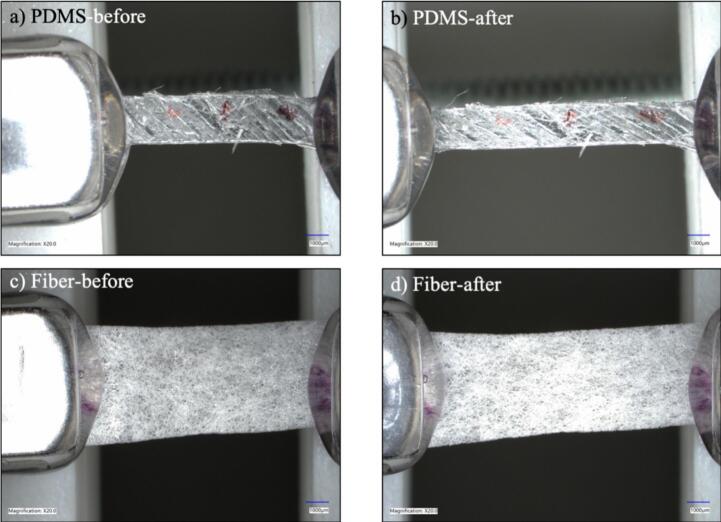
Demonstration under a Keyence microscope of sample stretched at 19 rpm for (a and b) PDMS and (c and d) electrospun fibers.

The clips were set to a starting distance of 10 mm, and each sample was stretched to 15 mm or 50 % strain at multiple strain rates (11 rpm, 15 rpm, and 19 rpm) for each sample type. Stretching was recorded using two digital microscopes: Keyence and Dinolite. Photos were taken before and after stretching on each microscope, with sample images recorded on Keyence shown for 11 rpm (Fig. 13) and 19 rpm (Fig. 14). Additionally, pictures and video-recorded stretch test data are available at the database location of Table 1. Testing showed that the hardware was able to successfully stretch a variety of sample types, while allowing for live imaging and video analysis.

## 8 Summary and conclusion

Dynamic mechanical analysis at the small scale is crucial for materials across a wide range of applications. In this paper, we developed a simple, affordable, easily reproducible, and compact microstretcher to enable such dynamic analysis. These qualities, in addition to being open source, make this device easily accessible to researchers across all backgrounds. The system can report strain values reliably and can be programmed to stretch samples at a designed strain rate under static or cyclic deformation, while remaining compact to fit into a variety of microscopes and spectroscopes. The device is designed to be easily modifiable after production; it remains stable and secure during measurement and live imaging analysis but can be disassembled and easily modified following experimentation to accommodate different sample requirements.

The device’s performance was validated using a variety of samples and microscope tests, with sample deformation captured in real time. During testing, slipping of the sample from the clips was not observed in either sample type. However, as is common amongst stretching devices, slipping from the clips could occur. To combat this slipping, clips with plastic covers were utilized to reduce the likelihood of slippage. If slipping occurs, sandpaper can be glued to the clips to increase friction and reduce the risk of slipping. If adding sandpaper does not prevent slipping, since the clips are low-cost and attached to removable clip boxes, the sample can be secured to the clips with glue. The clip boxes and clips would then need to be removed and replaced following each test. The total cost of filament for printing both clip boxes is $0.11, and the total cost of two clips is $0.59. Device compliance requires samples of specific geometries, mainly a sample width of 5 mm, a sample length of 30 mm or less, and a sample thickness of 5 mm or less. If the sample geometry is longer than 30 mm, the CAD file for the base could be altered to accommodate this increased length. Additionally, if the sample geometry is wider than 5 mm, wider clips could be purchased and attached to the clip boxes to accommodate this change.

The current limitation of the device is that it does not measure force values. Since the design is open source, future work and other researchers can expand upon it to include a force sensor to record and report force and deformation together. Overall, this device’s key advantages lie in its cost-effectiveness, ease of manufacturing, ease of use, and ease of modification. This open-source design will enable researchers to conduct static and dynamic micro-sample analysis on a broad spectrum of soft materials for various applications.

## 9 Human and Animal Rights

Not Applicable.

## CRediT authorship contribution statement

**Alexi Switz:** Writing – original draft, Visualization, Validation, Software, Methodology, Formal analysis. **Anamika Prasad:** Writing – review & editing, Supervision, Resources, Project administration, Funding acquisition, Conceptualization.

## Declaration of competing interest

The authors declare that they have no known competing financial interests or personal relationships that could have appeared to influence the work reported in this paper.
